# Getting Connected: a Retrospective Cohort Investigation of Video-to-Home Telehealth for Mental Health Care Utilization Among Women Veterans

**DOI:** 10.1007/s11606-022-07594-2

**Published:** 2022-08-30

**Authors:** Jan A. Lindsay, Alexandra Caloudas, Julianna Hogan, Anthony H. Ecker, Stephanie Day, Giselle Day, Samantha L. Connolly, Hilary Touchett, Kendra R. Weaver, Amber B. Amspoker

**Affiliations:** 1 VA South Central Mental Illness Research, Education and Clinical Center, a virtual center, Houston, TX USA; 2 Houston VA HSR&D Center for Innovations in Quality, Effectiveness and Safety, Michael E. DeBakey VA Medical Center, Houston, TX USA; 3 Baylor College of Medicine, Houston, TX USA; 4 Center for Healthcare Organization and Implementation Research, VA Boston Healthcare System, Boston, MA USA; 5 Harvard Medical School, Boston, MA USA; 6 Clinical Operations, Veterans Health Administration Office of Mental Health and Suicide Prevention, Washington, DC USA

**Keywords:** health services, women, telemedicine, veterans, mental health

## Abstract

**Background:**

Increasingly, women are serving in the military and seeking care at the Veterans Health Administration (VHA). Women veterans face unique challenges and barriers in seeking mental health (MH) care within VHA. VA Video Connect (VVC), which facilitates video-based teleconferencing between patients and providers, can reduce barriers while maintaining clinical effectiveness.

**Objective:**

Primary aims were to examine gender differences in VVC use, describe changes in VVC use over time (including pre-COVID and 6 months following the beginning of COVID), and determine whether changes over time differed by gender.

**Design:**

A retrospective cohort investigation of video-to-home telehealth for MH care utilization among veterans having at least 1 MH visit from October 2019 to September 2020.

**Participants:**

Veterans (236,268 women; 1,318,024 men).

**Interventions (if applicable):**

VVC involves face-to-face, synchronous, video-based teleconferencing between patients and providers, enabling care at home or another private location.

**Main Measures:**

Percentage of MH encounters delivered via VA Video Connect.

**Key Results:**

Women veterans were more likely than men to have at least 1 VVC encounter and had a greater percentage of MH care delivered via VVC in FY20. There was an increase in the percentage of MH encounters that were VVC over FY20, and this increase was greater for women than men. Women veterans who were younger than 55 (compared to those 55 and older), lived in urban areas (compared to those in rural areas), or were Asian (compared to other races) had a greater percentage of MH encounters that were VVC since the start of the pandemic, controlling for the mean percentage of VVC MH encounters in the 6 months pre-pandemic.

**Conclusions:**

VVC use for MH care is greater in women veterans compared to male veterans and may reduce gender-specific access barriers. Future research and VVC implementation efforts should emphasize maximizing patient choice and satisfaction.

A well-documented increase in women in the US military is resulting in growing numbers of women seeking health care from the Veterans Health Administration (VHA).^1^ From 2000 to 2015, women veterans choosing to receive care from VHA nearly tripled, with 22% currently engaged in care.^2^ Among women VHA users in 2015, mental health (MH) and substance use disorders represented the third most frequently diagnosed condition; and women VHA consumers with MH diagnoses quadrupled from 2000 to 2015.^2^ Women veterans differ from men in important ways. Demographically, they are more likely than men to be young and single, and identify as a racial or ethnic minority.^3,4^ Compared to men, women veterans are also at increased risk of posttraumatic stress disorder (PTSD), lifetime depression, and suicidal ideation.^3,4^ While nearly 40% of women veterans report military sexual trauma and over 50% report experiencing harassment during military service, women are more likely than men to perceive lower levels of unit support while in the military.^3,5^

Although women veterans evaluate MH care services as important, particularly for depression and PTSD, less than half (48.8%) treated at VHA reported their MH care needs were met completely or very well.^6,7^ The traditionally male-dominated VHA environment may make accessing MH care more difficult for women, particularly survivors of sexual trauma.^8^ Approximately 25% of women veterans report experiencing gender-based harassment at VHA (e.g., mixed-gender waiting rooms), and most have witnessed harassment there.^9,10^ Some women may delay care or seek treatment at women’s clinics and/or from women providers to avoid environmental challenges.^10,11^ Limited availability of providers with women’s health training and lack of gender-specific services are significant barriers for women veterans at some VHA sites and may be a greater barrier for women identifying as racial, ethnic, or sexual minorities.^6,7^

Women veterans also face logistical barriers to accessing MH treatment, including treatment cost, stigma, difficulty finding transportation or childcare/eldercare, and inability to take time from work.^11–13^ Recent data highlight the significant and unequal childcare burden of working women with young children, which may negatively impact women’s MH.^14^ In a recent qualitative study, VHA stakeholders cited childcare issues as distinct access barriers for women.^11^ Rural women veterans may also have increased travel distance and limited availability of specialty care providers.^15,16^

VHA became an early adopter of telehealth.^17,18^ Its preferred platform, VA Video Connect (VVC), involves face-to-face, synchronous, video-based teleconferencing between patients and providers, enabling care at home or another private location. VVC can reduce barriers for women veterans by facilitating access to providers with women’s health expertise, eliminating geographical barriers, and increasing convenience for caregivers for family members.^19,20^ Remotely delivered care is equally effective as in-person care for many MH concerns, including PTSD and depression, and is associated with high patient and provider satisfaction.^20–24^ While the pandemic prompted an unprecedented rise in telehealth appointments and reduced in-person healthcare visits nationally, the prevalence of MH concerns significantly increased.^25,26^ Our primary aims were to 1) examine differences in VVC use by gender, 2) describe changes in VVC use over time (including pre-COVID pandemic and 6 months following beginning of the pandemic), and 3) determine whether changes over time differed by gender. To better understand gender differences in remotely delivered MH care utilization among women veterans, we examined and compared patterns in VVC use for MH care among men and women veterans utilizing VHA healthcare. We also examined differences in women veterans’ VVC use by race, age, and rurality. Health disparities by race are well-documented, and members of racial and ethnic minorities may have less access to internet and video-enabled devices than White Americans, limiting access to tech-based interventions like VVC.^27,28^ Furthermore, research documents MH treatment barriers for older adults and rural individuals.^16,29^ We hypothesized that (a) women veterans are more likely to have engaged in MH care via VVC; (b) women veterans have a greater percentage of MH care that is VVC; (c) differences in the percentage of pre-pandemic VVC use between men and women veterans would persist throughout pandemic months; (d) gender differences in percentage of one’s MH care that is VVC would persist regardless of differences in age, rurality, race, and medical comorbidities; and (e) VVC use would be highest among women veterans who were younger (<55), urban, and identifying as non-Hispanic White.

## METHODS

A retrospective cohort investigation of video-to-home telehealth for MH care utilization was conducted using data from the Corporate Data Warehouse. A national cohort of patients receiving VHA care was identified by selecting veterans completing at least 1 MH encounter during Fiscal Year 2020 (October 2019–September 2020). MH clinic stop codes (500 series) were used to identify MH services accessed by patients, with a 179 secondary stop code to capture VVC visits to a non-VHA location. The Deyo-Charlston comorbidity index was calculated for each veteran, using ICD10 codes. Demographic data, including gender, age, race, and rurality, collected via veteran self-report during VHA enrollment was also obtained. Mode of delivery for MH encounters included VVC, in-person, and telephone. The final cohort included 12 months’ data for all veterans (236,268 women: 1,318,024 men). This study was approved by the Institutional Review Boards of participating institutions.

### Statistical Analyses

We examined differences between veteran women and men in demographic characteristics using an independent samples *t*-test (for age) and chi-square tests (for rurality and race/ethnicity).

We then examined gender differences in VVC MH encounters in FY20. Two dependent variables were evaluated. First, we examined gender differences in the likelihood of at least 1 VVC MH encounter in FY20. We conducted a logistic regression model, with the outcome being whether there was a VVC MH encounter at any point during FY20 (where 0 = no and1 = yes), and the independent variable was gender (where 0 = men and 1 = women). Second, we examined gender differences in the percentage of MH encounters delivered via VVC (i.e., number of VVC encounters for MH divided by all MH encounters) monthly across FY20, using linear growth curve models. The dependent variable was the percentage of MH encounters that were VVC (from 0 to 100%). The first model included gender and time (FY20, where October 2019 was coded as 0, November 2019 as 1, and so forth, all the way to September 2020, coded as 11) as fixed independent variables and enabled examination of differences in the percentage of MH encounters that were VVC by gender over time. A second model also included the interaction between gender and time as a fixed independent variable to examine whether change over time differed by gender. Intercept and time were included as random effects in all growth-curve models. We subsequently conducted similar analyses separately among pre-pandemic months (October 2019–February 2020) and during the first 6 full months of the pandemic (April 2020–September 2020).

Finally, women veteran-only subgroup analyses were conducted to investigate age, rurality, and ethnic/racial differences in percentage of VVC MH encounters since the pandemic started. We conducted 3 analysis of covariance (ANCOVA) models in which the average percentage of MH encounters that were VVC in August and September 2020 was the dependent variable and age (less than 55 versus 55 and older), rurality (highly rural/rural versus urban), and race/ethnicity were independent variables. Each model controlled for the average percentage of VVC MH encounters in the 6 months before the pandemic. Since not every veteran had an MH encounter in August and/or September of 2020, analyses were repeated using PROC MI and MIANALYZE intent-to-treat procedures in SAS. Fully adjusted models were then examined, such that models were repeated with inclusion of age category (less than 55 versus greater than or equal to 55), rurality (rural vs urban), race/ethnicity (Asian, White Hispanic/Latino, Native Hawaiian/Pacific Islander, Black, Non-Hispanic White, and American Indian/Alaskan Native), and Deyo score as covariates. All statistical analyses were conducted using SAS Version 9.4 (SAS Institute, Cary, NC).

## RESULTS

**Figure 1:**
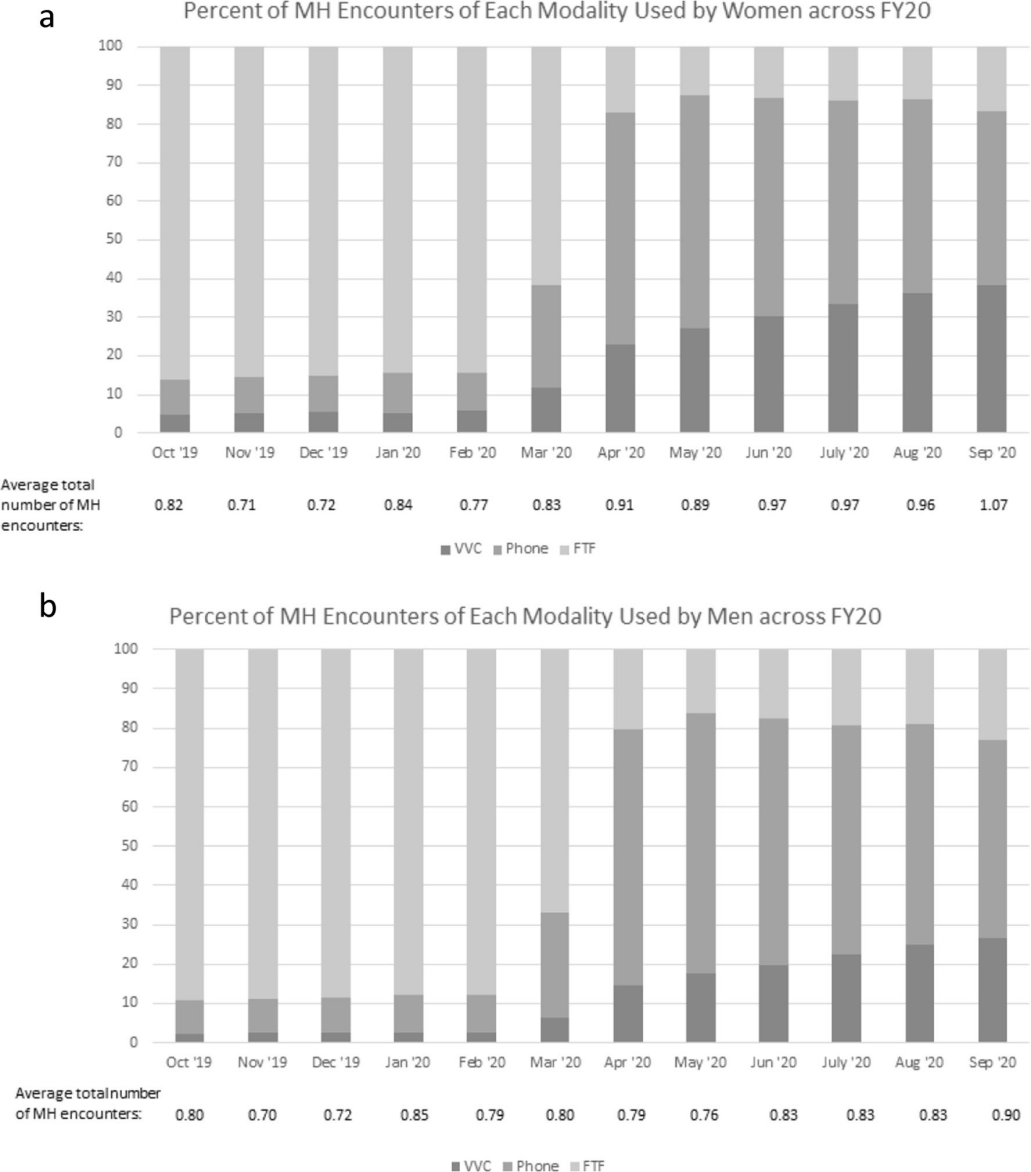
**a** The percentage of mental health encounters of each modality used by women across FY20. **b** The percentage of mental health encounters of each modality used by men across FY20.

Demographic characteristics of study participants overall and by gender are reported in Table 1. Compared to men, women veterans were significantly younger, more likely to be urban, and less likely to be non-Hispanic White. Although effect sizes for rurality and race/ethnicity were very small (*d*s = 0.04 and 0.10, respectively), the effect for age was medium-to-large (*d* = 0.62). Figure 1a and b report the average total number of MH encounters in each month of FY20 and depict the percentage of all encounters of each modality for veteran women and men, respectively.

**Table 1:** Demographics overall and for women versus men who had at least one mental health encounter between October 2019 and September 2020. N = 1,554,292 unless otherwise noted

	Total (N = 1,554,292)	Women (n = 236,268)	Men (n = 1,318,024)	*p* value	Effect size *d*/phi
Age, mean (SD)	54.65 (16.06)	46.79 (13.52)	56.02 (16.07)	<0.0001	0.62
Rurality, N (%) (n = 1,503,397)				<0.0001	0.04
Urban	1,075,526 (71.53)	173,300 (76.10)	902,226 (70.72)		
Rural	415,283 (27.62)	53,064 (23.30)	362,219 (28.39)		
Highly Rural	12,688 (0.84)	1,376 (0.60)	11,312 (0.89)		
Race/Ethnicity N (%) (n = 1,518,057)				<0.0001	0.10
Black	362,362 (23.87)	77,025 (33.66)	285,337 (22.13)		
White – Non-Hispanic	936,331 (61.68)	117,987 (51.55)	818,344 (63.48)		
White – Hispanic/Latino	163,684 (10.78)	23,941 (10.46)	139,743 (10.84)		
Asian	20,298 (1.34)	3,624 (1.58)	16,674 (1.29)		
American Indian /Alaskan Native	17,489 (1.15)	3,265 (1.43)	14,224 (1.10)		
Native Hawaiian /Pacific Islander	17,893 (1.18)	3,024 (1.32)	14,869 (1.15)

### Gender Differences in VVC Use for MH Care During Fiscal Year 2020

Among those with MH encounters during FY20, women veterans were 77% more likely to have had at least 1 VVC encounter than men (95% CIs = 1.76 and 1.77), *χ*^2^(1) = 53816.74.

**Figure 2:**
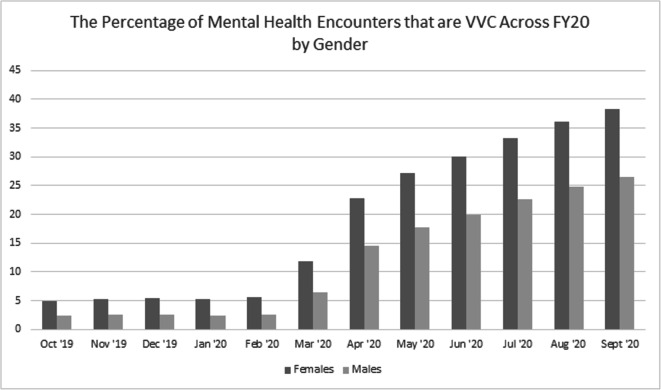
The percentage of mental health encounters that are VVC across FY20 by gender. VVC, VA Video Connect.

See Figure 2 for the mean percentage of MH encounters that were VVC over time by gender. In FY20, there was a main effect of gender (*b* = 3.87, *SE* = 0.04, *t* (16E5) = 99.12, *p* < 0.0001) and a main effect of time (*b* = 2.43, *SE* = 0.004, *t*(54E5) = 590.78, *p* < 0.0001). Women veterans had nearly 4% more VVC MH encounters than men. Furthermore, there was a 2.43% increase per month in percentage of VVC MH encounters. Importantly, there was a significant interaction between gender and time (*b* = 0.95, *SE* = 0.01, *t*(54E5) = 84.95, *p* < 0.0001), such that the effect of time for women (*b* = 3.28, *SE* = 0.01, *t*(91E4) = 314.31, *p* < 0.0001) was greater than the effect of time for men (*b* = 2.28, *SE* = 0.004, *t*(45E5) = 577.62, *p* < 0.0001). Subsequent analyses for the pre-pandemic and pandemic periods revealed similar findings (see Table 2). Importantly, all findings persisted, controlling for age category, race/ethnicity, rurality, and the Deyo-Charlston comorbidity index.

**Table 2:** Results of the Individual Growth Curve Models Predicting the Percentage of Mental Health Encounters Conducted via VVC Both Pre-COVID-19 and Since COVID-19

Effect	PreCOVID-19 (October 2019–February 2020)		Since COVID-19 (April 2020–September 2020)	
	*b* (SE)	*p*-value	*b* (SE)	*p*-value
Time	2.43 (0.004)	<0.0001	2.40 (0.04)	<0.0001
Gender (reference group = men)	3.35 (0.04)	<0.0001	9.02 (0.08)	<0.0001
Time x Gender Interaction	0.95 (0.01)	<0.0001	0.73 (0.03)	<0.0001

For both the pre-COVID-19 and since COVID-19 analyses, the first model contained the main effects of time and gender. The second model contained the same effects with the addition of the interaction between time and gender

*VVC*, VA Video Connect

### An Examination of Age, Rurality, and Race in Women Veterans Only

Among women veterans, age category, rurality, and race/ethnicity each significantly predicted percentage of VVC MH encounters 6 months after the pandemic started (see Table 3). Specifically, the percentage of VVC MH encounters 6 months after the pandemic began was significantly lower for rural women veterans (*F*(1, 103456) = 187.39, *p* < 0.0001) and those at least 55 years of age (*F*(1,103491) = 1222.27, *p* < 0.0001). Furthermore, there were significant racial/ethnic differences in percentage of VVC MH encounters, *F*(5, 108314) = 63.28, *p* < 0.0001. Asian women veterans had a significantly greater percentage of VVC encounters than all other races/ethnicities (all *p*s < 0.01). Conversely, American Indian/Alaskan Native and non-Hispanic White women veterans had a significantly lower percentage of VVC MH encounters than all other races/ethnicities (all *p*s < 0.05). Intent-to-treat analyses and fully adjusted models revealed parallel findings.

**Table 3:** Adjusted Means from ANCOVA Models That Examined Differences in the Percentage of Mental Health Encounters Conducted via VVC 6 Months After the Pandemic Began by Age, Rurality, and Race/Ethnicity *For Women Veterans Only*

	Adjusted mean percentage of mental health encounters conducted via VVC 6 months after the COVID-19 pandemic began
Age	
<55 years of age	35.94^a^
≥ 55 years of age	26.52^b^
Rurality	
Rural/Highly Rural	31.74^a^
Urban	35.82^b^
Race/ethnicity	
Asian	42.44^a^
White Hispanic/Latino	39.25^b^
Native Hawaiian/Pacific Islander	38.38^b^
Black	35.62^c^
Non-Hispanic White	33.05^d^
American Indian/Alaskan Native	32.53^d^

Means are adjusted for the percentage of mental health encounters that are VVC in the 6 months prior to the pandemic. Within each age, rurality, and race/ethnicity, categories with different subscripts differ from each other at *p* < 0.05

## DISCUSSION

Women veterans in our sample received MH care via VVC at significantly higher rates than male veterans. Although overall rates of VVC use increased for both genders, women’s use increased more steeply over time. Importantly, observed gender differences in VVC use existed before and persisted throughout the first 6 months of the pandemic, indicating that women veterans’ preference for VVC was not entirely explained by efforts to prevent COVID-19 infection. Women veterans in our sample were younger and less likely to be non-Hispanic White, consistent with other findings that women veterans are more likely than men to be young and identify as a racial or ethnic minority.^4^ Interestingly, gender differences in VVC use remained after controlling for age, race/ethnicity, rurality, and medical comorbidities, suggesting that women veterans’ higher rates of VVC use cannot be explained by demographic differences between the 2 groups.

Several factors could be driving gender differences. In a recent review, women experienced significantly fewer user issues with telepsychiatry than men, suggesting they may have increased comfort or skill navigating telehealth.^30^ Additionally, a study investigating women veterans’ preferences for MH treatment delivery revealed that over half preferred to receive treatment at home (e.g., telehealth-to-home or in-person treatment at home),^31^ some due to caregiving responsibilities and challenges associated with leaving home for treatment.^20^ These findings are consistent with research in the general population indicating that women are more likely than men to prefer, use, and report being satisfied with telehealth for MH.^32–35^

Another potential explanation for higher rates of VVC use among women veterans relates to their VHA experiences. As noted, women veterans frequently feel unwelcome and harassed while at VHA (e.g., while in mixed-gender settings), and remotely delivered MH treatments may increase their comfort.^10,36^ For women veterans who lack access to on-site providers with expertise in women’s health, especially those in rural settings, VVC can facilitate greater access to gender-specific services and specialty providers.^11,19^ Importantly, having access to women providers and women-only treatment groups is associated with perceived increased access to MH services, demonstrating that treatment experiences may influence perceptions of overall access to care.^7^

Gender differences in VVC use could also be partially driven by patient and provider preferences for modality, as well as type of treatment offered and/or sought. For example, providers may systematically offer women veterans video visits at higher rates than men; or women may request VVC more often than men. They may also partly depend on the type of MH appointments veterans are receiving. Men and women differ significantly in MH care preferences (i.e., psychotherapy versus medication management), with women more likely to choose psychological treatment than medication management.^37^ Given that medication management psychiatric appointments are typically shorter than psychotherapy sessions, MH providers prescribing pharmacological treatment may have used telephone calls rather than VVC; however, this requires follow-up analyses.

Although women veterans engaged in MH care via VVC at greater rates than men veterans, disparities in VVC use emerged within our sample, based on age, race, and rurality. The percentage of VVC MH encounters was significantly lower for women veterans aged 55 or older, indicating that older women veterans were less likely than younger counterparts to receive MH treatment via VVC. Several factors could be responsible. Comfort with and access to technology may vary by age, with younger adults using more technological tools daily.^38^ Inexperience with technology may be a barrier to accessing MH care delivered via technological innovations.^39^ Recent evidence suggests a “grey digital divide,” that individuals over 55 may be less likely than younger individuals to have internet access and complete telemedicine visits.^40,41^ Older adults tend to be under-consumers of MH treatment; and they are less likely than younger adults to be engaged in psychological treatment for MH concerns.^29,37^ This comparative lack of engagement in psychological therapies by older adults may be due to patient preferences and provider beliefs about older adults’ receptivity to psychological intervention.^37,42^

Additional differences among women veterans’ VVC use were observed by rurality and race. Rural women veterans were less likely to receive VVC for MH treatment, consistent with literature documenting disparities in access to MH treatment in rural areas and slower adoption of innovative approaches to treating MH concerns.^43,44^ Moreover, contrary to hypotheses, Asian American women veterans had a significantly greater percentage of VVC MH appointments than all other racial and ethnic groups including women veterans identifying as non-Hispanic White. During the pandemic, Asian Americans experienced significant levels of racism and discrimination; and fear of discrimination may have caused Asian American women veterans to seek remotely delivered care over in-person treatment.^45^ In contrast, American Indian and Alaskan Native women veterans had a significantly lower percentage of VVC encounters 6 months after the pandemic began compared to all other racial and ethnic groups. American Indian and Alaskan Native communities were significantly impacted by the pandemic, experiencing 3.5 times greater risk of being diagnosed with COVID-19 and a mortality rate twice that of non-Hispanic White Americans.^46^ American Indian and Alaskan Native communities lack equitable access to MH treatment, and lack of access to technology is a barrier to receiving remotely delivered MH treatment.^47,48^

This project should be viewed in the context of its limitations. Although this database offers important information regarding veterans’ MH diagnoses and access to treatment using VVC, it does not furnish information on frequencies of non-VVC-related treatment experiences. Additionally, the data do not explain why veterans received MH treatment via VVC, including if and/or how VVC utilization was driven by veteran and provider factors or treatment type. It also does not provide information on whether an interaction exists between gender and clinic type. In other words, it does not explain if women were more likely to seek care from clinics with higher VVC adoption or if certain women’s clinics offering MH care were more likely to use VVC. While the data do not offer guidance on whether variations in VVC adoption exist by geographical location, we do not anticipate significant differences between regions due to the large integrated nature of the VA medical system coupled with the heterogeneity of both rural and urban sites within each region. Finally, although the data reveal a steep increase in VVC use in the 6 months following declaration of the pandemic, it is unknown how VVC use will evolve in a post-COVID world and whether the observed increase will continue. Future research should investigate factors predicting VVC use compared to other modalities of care, such as patient and provider preferences, clinic and/or treatment type, digital health literacy, and barriers to in-person care (e.g., geographical limitations). It should also examine whether an interaction exists between gender, treatment type, and modality (in-person care, telehealth, etc.).

The COVID-19 pandemic dramatically changed healthcare delivery. Although telehealth’s role in post-pandemic life remains unclear, patient preferences are important. According to a 2021 poll by the American Psychological Association, nearly half of Americans feel concerned about returning to in-person activities, regardless of COVID-19 vaccination status.^49^ Others may prefer telehealth appointments. Although variability exists in patient circumstances and preferences, VVC is an important part of patient-centered care for women veterans. Future research and VVC implementation efforts should emphasize maximizing patient choice and increasing veterans’ satisfaction with MH care.
